# Malic Enzyme 2 Regulates Dynamin-Related Protein 1-Dependent Mitochondrial Fission and Mitochondria-Associated Membranes to Drive Odontogenic Differentiation: An In Vitro and In Vivo Study

**DOI:** 10.3390/biom16050664

**Published:** 2026-04-30

**Authors:** Jingzhou Li, Qianyi Shi, Xinyue Sheng, Haozhen Ma, Qianyi Deng, Yifan He, Fuping Zhang, Fang Huang

**Affiliations:** 1 Hospital of Stomatology, Sun Yat-sen University, Guangzhou 510055, China; lijzh5@mail2.sysu.edu.cn (J.L.); shiqy8@mail2.sysu.edu.cn (Q.S.); shengxy@mail2.sysu.edu.cn (X.S.); mahzh5@mail2.sysu.edu.cn (H.M.); dengqy29@mail.sysu.edu.cn (Q.D.); heyf59@mail.sysu.edu.cn (Y.H.); 2 Guangdong Provincial Key Laboratory of Stomatology, Guangzhou 510080, China

**Keywords:** malic enzyme 2, dynamin-related protein 1, mitochondrial fission, mitochondria-associated endoplasmic reticulum membranes, dental papilla cells, odontogenic differentiation

## Abstract

The differentiation of dental papilla cells (DPCs) into functional odontoblasts is critical for dentinogenesis, yet the role of mitochondrial dynamics remains unclear. Here, we investigated the functional role of mitochondrial fission and mitochondria-associated endoplasmic reticulum membranes (MAMs) in the odontogenic differentiation of DPCs. Using in vitro differentiation models combined with confocal microscopy, transmission electron microscopy, and gain- and loss-of-function approaches, we found that odontogenic induction triggered early mitochondrial fragmentation and increased MAM formation. Dynamin-related protein 1 (DRP1) mediated mitochondrial fission, which in turn regulated MAM architecture and promoted differentiation. Malic enzyme 2 (ME2) acted as an upstream regulator, facilitating DRP1 recruitment and organizing MAM integrity. Notably, disruption of the ME2-DRP1-MAM axis impaired dentin formation both in vitro and in vivo, either by ME2 knockdown or pharmacological inhibition of DRP1 (Mdivi-1). These findings establish the ME2-DRP1-MAM axis as a critical metabolic–organellar switch driving odontoblast differentiation, providing new mechanistic insights into dentinogenesis and identifying potential therapeutic targets for dentin–pulp complex regeneration.

## 1. Introduction

Dental papilla cells (DPCs), the mesenchymal progenitors of odontoblasts, hold significant promise in dental regenerative medicine due to their multidirectional differentiation potential [1,2,3]. The process of odontogenic differentiation is orchestrated by a complex regulatory network that remains only partially understood. While transcription factors and signaling pathways have been extensively studied, emerging evidence suggests that cellular metabolism [4] and organelle dynamics [5] play equally important but under-explored roles in directing cell fate decisions.

Among intracellular organelles, mitochondria are increasingly recognized as dynamic signaling hubs, with their continuous fission and fusion being critical for functional homeostasis [6]. A shift towards fission serves as an early and essential event in the differentiation of various stem cell lineages, facilitating metabolic reprogramming and biomass distribution [7]. The core machinery of mitochondrial fission involves the translocation of dynamin-related protein 1 (DRP1) from the cytosol to the mitochondrial outer membrane, a process primarily mediated by the receptor mitochondrial fission factor (MFF) [8]. Upon recruitment, DRP1 oligomerizes into ring-like structures that constrict and ultimately sever mitochondrial tubules to complete fission [9]. DRP1-mediated mitochondrial fission is well established in driving mesenchymal stem cell (MSC) differentiation [10] and is important for embryonic and neuronal development [11,12]. However, current evidence remains conflicting regarding its role in tooth development. Under physiological conditions, fission-mediated mitochondrial remodeling promotes differentiation of dental pulp cells [13], whereas under high-glucose stress or inflammatory aging, excessive fission triggers mitochondrial dysfunction and apoptosis [14]. This context-dependent effect explains the apparent discrepancy in the literature. Elucidating its role in dentin formation thus has clinical implications for dentin regeneration and theoretical significance for organ development.

Notably, approximately 90% of mitochondrial fission events occur at specialized contact sites between mitochondria and the endoplasmic reticulum (ER), known as mitochondria-associated ER membranes (MAMs) [15]. These domains, with a width of 10–30 nm, are crucial for inter-organellar communication [16]. The recruitment and assembly of DRP1 at MAMs are key to initiating fission at these sites, with MFF being a critical recruiter [17]. While MAMs have been implicated in facilitating mitochondrial transfer and repair in dental pulp stem cells [18], their specific role, in concert with mitochondrial dynamics, in driving odontogenic differentiation remains a significant knowledge gap.

Malic enzyme 2 (ME2), a NAD+-dependent enzyme that catalyzes the conversion of malate to pyruvate in the mitochondrial matrix, is traditionally viewed as a key metabolic enzyme. However, recent studies hint at its potential non-metabolic functions, including the regulation of mitochondrial dynamics. Our previous study demonstrated that ME2 upregulates the expression of optic atrophy 1 (OPA1), mitofusin 2 (MFN2), thereby promoting mitochondrial fusion and enhancing aerobic respiration. This process, in turn, drives the differentiation of DPCs, suggesting a critical role for ME2 in dentin formation [19]. Notably, mitochondrial fission and fusion are not opposing forces. Instead, the continuous cycles of fission and fusion regulate nearly every aspect of mitochondrial physiology [20]. We thus hypothesize that a regulatory axis involving DRP1, MAMs and ME2 may exist to coordinate mitochondrial function and dynamics, thereby driving the odontogenic differentiation of DPCs.

This study aims to elucidate the role of the ME2-DRP1-MAM axis in mediating mitochondrial fission during the odontogenic differentiation of DPCs. We hypothesize that ME2 regulates MAM formation by promoting DRP1-dependent mitochondrial fission, thereby driving DPC differentiation and dentin formation. Elucidating this pathway will provide novel mechanistic insights into dentinogenesis and open new avenues for regenerative tooth engineering.

## 2. Materials and Methods

### 2.1. Primary Culture of DPCs

These animal experiments were approved by the Institutional Animal Care and Use Committee of Sun Yat-sen University (SYSU-IACUC-2025-002078). DPCs were cultured from dental papilla tissue isolated from postnatal day 1–3 (PN1–3) Sprague-Dawley (SD) rats. The molar tooth germs were dissected according to an established protocol under a stereomicroscope (Carl Zeiss AG, Oberkochen, Germany) [21]. The tissues were digested for 15 min at 37 °C in a solution of 3 mg/mL collagenase I and 4 mg/mL dispase II (Sigma-Aldrich, St. Louis, MO, USA). The digested tissue was resuspended in primary medium: α-MEM (Gibco, Grand Island, NY, USA) supplemented with 15% FBS (Cyagen, Santa Clara, CA, USA), 100 U/mL penicillin, 100 µg/mL streptomycin, and 100 µg/mL glutamine (all from Gibco). Cells were plated and maintained at 37 °C with 5% CO_2_. Upon reaching 80–90% confluence, they were passaged using TrypLE (Gibco) and subsequently cultured in basal growth medium (Ctrl, α-MEM with 8% FBS and antibiotics/glutamine). Cells from passages 3–4 were used for all experiments.

### 2.2. Odontoblastic Differentiation and Adipogenic Differentiation

For odontogenic differentiation, DPCs were cultured for 7 days in osteogenic/odontogenic medium (OS), which consisted of α-MEM supplemented with 8% FBS, 0.2 mM ascorbic acid, 10 nM dexamethasone, 10 mM β-glycerol phosphate (all from Sigma-Aldrich), and standard antibiotics/glutamine.

For adipogenic differentiation, cells were induced for 14 days in basal medium supplemented with 1 µM dexamethasone, 200 µM indomethacin, 0.5 mM IBMX, and 10 µg/mL insulin (all from Sigma-Aldrich). Lipid droplets were detected by staining with Oil Red O solution (Cyagen) according to the manufacturer’s protocol, observed and photographed under an inverted microscope (Carl Zeiss AG).

### 2.3. Colony-Forming Unit Assay

DPCs in good condition were seeded at a low density of 1000 cells per 6-cm^2^ dish and cultured for 14 days. The cells were then fixed, stained with 0.1% crystal violet for 20 min, followed by washing with PBS (Gibco). Colonies were examined and documented using an inverted microscope (Carl Zeiss AG).

### 2.4. Alizarin Red Staining (ARS)

Following fixation (4% paraformaldehyde, 30 min), cells were stained with Alizarin Red S solution (Cyagen; pH 4.2, 30 min, dark). Plates were scanned and imaged under an inverted microscope (Olympus IX83, Olympus Corporation, Tokyo, Japan). Mineralized nodules were solubilized in 100 mM cetylpyridinium chloride (Sigma-Aldrich) and quantified by absorbance at 562 nm (Epoch2 microplate reader, Biotek, Winooski, VT, USA).

### 2.5. Flow Cytometry

DPCs were analyzed by flow cytometry for mesenchymal stem cell (MSC) marker expression. Single-cell suspensions were prepared, filtered through a 70 μm mesh, and adjusted to 5 × 10^6^ cells/mL in 1% BSA/PBS after centrifugation. The suspension was individually stained with FITC- or PE-conjugated antibodies against CD29, CD44, CD90, CD34, and CD45, along with matched isotype controls (Elabscience, Wuhan, China), for 30 min in the dark at room temperature. Subsequently, the stained cells were washed, resuspended in PBS, and analyzed by flow cytometry (Beckman Coulter, Indianapolis, IN, USA).

### 2.6. Mitochondrial and ER Staining

For mitochondrial and cytoskeletal staining, cells cultured on glass-bottom dishes for 5 days were incubated with 200 nM MitoTracker Red CMXROS (Beyotime, Shanghai, China) in α-MEM at 37 °C for 30 min, washed, and fixed with 4% formaldehyde. Actin filaments were labeled with Actin-Tracker Green-488 (1:100, Beyotime) for 30 min. Nuclei were counterstained with DAPI, and images were acquired using a confocal microscope (Olympus FV3000, Olympus Corporation).

For mitochondria–ER co-staining, cells were rinsed with warm HBSS (Gibco) and sequentially incubated with 1 μM ER-Tracker Green (Thermo Fisher Scientific, Waltham, MA, USA) and 200 nM MitoTracker Red CMXROS for 30 min at 37 °C. After washing, cells were fixed with absolute ethanol (5 min), and nuclei were stained with DAPI. Images were acquired immediately by confocal microscopy (Olympus FV3000, Olympus Corporation).

For each coverslip, 5–10 fields containing well-separated, intact cells were selected for imaging. At least 10 cells per group were analyzed. Image quantification was performed blinded to the experimental groups.

### 2.7. Western Blot

Cell total protein was extracted with RIPA lysis buffer (Beyotime) comprising 1% protease and phosphatase inhibitors (PMSF, Beyotime). After centrifugation (14,000× *g*, 20 min, 4 °C), protein concentration was measured with a BCA kit (CWBio, Beijing, China). Equal amounts (20 µg) of protein were mixed with 5× Loading Buffer (CWBio), denatured, separated on 4–12% SDS-PAGE gels (ACE Biotechnology, Changzhou, China), and transferred to 0.45 µm PVDF membranes (EMD Millipore, Billerica, MA, USA) in a wet tank system (200 mA, 90 min, 4 °C). The membranes were blocked in 5% skimmed milk for 1 h, incubated overnight at 4 °C with primary antibodies, then with HRP-conjugated secondary antibodies for 1 h at room temperature. Bands were visualized by ECL (EMD Millipore) with a ChemiDoc system (Bio-Rad, Hercules, CA, USA), and densitometry was performed with ImageJ 1.54g software (NIH, Bethesda, MD, USA). Antibody details are listed in Table 1.

### 2.8. Real-Time Quantitative Polymerase Chain Reaction (RT-qPCR)

Total RNA from DPCs was extracted with the RNA-Quick Purification Kit (Yishan Biotechnology, Shanghai, China) according to the manufacturer’s instructions. RNA purity and concentration were assessed using a NanoDrop spectrophotometer (NanoDrop Technologies, Wilmington, DE, USA). First-strand cDNA was generated with PrimeScript RT Master Mix (TaKaRa, Shiga, Japan) and subjected to qPCR using SYBR Green (Yeasen, Shanghai, China) with gene-specific primers listed in Table 2 (RuiBiotech, Guangzhou, China). The β-actin gene served as the internal reference, and relative expression levels were quantified via the 2^−ΔΔCt^ method.

### 2.9. Transmission Electron Microscopy (TEM)

Cell pellets were fixed overnight in 2.5% glutaraldehyde (4 °C), post-fixed with 1% osmium tetroxide (1.5 h), and stained with 3% uranyl acetate (2 h). Following ethanol dehydration and Epon 812 (all from Servicebio, Wuhan, China) embedding, ultrathin sections (≈50 nm) were collected on carbon-coated grids, contrasted with 0.3% lead citrate, and examined under a transmission electron microscope (Hitachi H-7650, Tokyo, Japan).

MAMs were identified as regions where the distance between the outer mitochondrial membrane and the ER membrane was ≤30 nm, based on established criteria [15]. For TEM analysis, at least 15–20 random fields per sample were examined, with fields selected systematically by moving the stage in a predefined pattern (top-left to bottom-right) to avoid selection bias. At least 50 mitochondria per condition were analyzed from these fields. All quantifications were performed blinded to experimental conditions. The following parameters were quantified: (1) MAMs/mitochondria perimeter ratio: the length of MAMs divided by the mitochondrial perimeter; (2) ER–mitochondria distance: the average shortest distance between ER and mitochondrial membranes; (3) ER–mitochondria contact coefficient (ERMICC) was calculated as Interface length of ER/(Mitochondrial perimeter × ER–mitochondria distance) [22].

### 2.10. Immunofluorescence (IF)

Cells for immunofluorescence were seeded in 15 mm confocal dishes at a density of 1 × 10^4^ cells under optimal conditions for 5 days. Following a PBS rinse, cells were fixed, permeabilized, and blocked. They were subsequently incubated overnight at 4 °C with specific primary antibodies (anti-DRP1; details in Table 1). After incubation with fluorescently conjugated secondary antibodies (DyLight 488/594) in the dark, nuclei were counterstained with DAPI-containing mounting medium (Beyotime) and visualized by a laser confocal microscopy (Olympus).

At least 15–20 cells per condition (from at least three representative images) were analyzed for confocal imaging. Quantification of fluorescence intensity and co-localization was performed blinded using ImageJ, with background subtraction and normalization to the control group.

### 2.11. Small Interfering RNA (siRNA) Transfection

DPCs were seeded in 12-well plates at 1.5 × 10^5^ cells/well cultured for 24 h to reach 70–80% confluence before transfection. DRP1-targeting siRNA (si-DRP1) and negative control siRNA (si-NC) were designed and synthesized by Forevergen (Guangzhou, China, sequences in Table 3). For transfection, 2 µL siRNA and 2 µL Lipofectamine 3000 (Invitrogen, Waltham, MA, USA) were separately diluted in 100 µL Opti-MEM (Gibco), mixed, and incubated for 15 min at room temperature. The complexes were added dropwise to wells, containing fresh antibiotic-free medium (final siRNA concentration: 50 nM). Cells were cultured in OS medium for 5 days before harvesting. Knockdown efficiency was validated by RT-qPCR and Western blot.

### 2.12. Lentiviral Transduction Process

Lentiviruses to knock down ME2 (sh-ME2), overexpress ME2 (oe-ME2), and negative control (sh-NC, oe-NC) were synthesized by Hanbio (Shanghai, China). Lentivirus to overexpress DRP1 (oe-DRP1) and negative control (oe-NC) were synthesized by GeneCopoeia (Guangzhou, China). Then they were used per the recommended conditions with indicated multiplicity of infection (MOI: sh-ME2 = 20, oe-ME2 = 20, oe-DRP1 = 10). The shRNA targeting rat ME2 (RefSeq NM_001318900.1) is 5′-GAGGTCATGTTAGATCAATTG-3′. The negative control shRNA sequence is 5′-TTCTCCGAACGTGTCACGTAA-3′. Transduction efficiency was confirmed by GFP fluorescence microscopy (estimated >80% positive cells from five random fields per group), RT-qPCR, and Western blot.

### 2.13. Alkaline Phosphatase (ALP) Staining and Activity Detection

For ALP staining, cells were fixed in 4% paraformaldehyde for 30 min, rinsed three times with PBS, and stained with BCIP/NBT alkaline phosphatase substrate solution (Beyotime) for 30 min in the dark. Total protein was collected and ALP activity was detected by the ALP activity assay kit (NJJCbio, Nanjing, China) according to the manufacturer’s instructions.

### 2.14. Immunohistochemistry (IHC)

Mandibles from PN 3–5 SD rats were fixed in 4% paraformaldehyde for 24 h after euthanasia, decalcified in 10% EDTA for 7 days, and embedded in paraffin following gradient dehydration and clearing. Sections (4 μm) were dewaxed, rehydrated, and subjected to antigen retrieval by microwave heating in EDTA buffer. After blocking with BSA, sections were incubated with primary anti-DRP1 antibody (Table 1) overnight at 4 °C, followed by HRP-conjugated secondary antibody, DAB development, and hematoxylin counterstaining. Finally, sections were dehydrated, cleared in xylene, mounted, and scanned using a digital slide scanner (Aperio AT2; Leica, Wetzlar, Germany).

### 2.15. Ectopic Subcutaneous Transplantation Model

To evaluate the role of DRP1 in DPC-mediated odontogenesis in vivo, DPCs were transfected with si-NC or si-DRP1, or transduced with oe-NC or oe-DRP1, and resuspended in Matrigel (Corning, Corning, NY, USA) at 1 × 10^7^ cells/mL. A 200 μL aliquot of the cell–Matrigel mixture was injected subcutaneously into each 6-week-old BALB/c nude mouse. After 4 weeks, mice were euthanized via anesthetic overdose, and implants were harvested for histological analysis.

### 2.16. Histological Analysis

Paraffin-embedded sections were dewaxed, rehydrated, and subjected to hematoxylin and eosin (H&E) or Masson’s trichrome staining. For H&E, sections were stained with hematoxylin (Servicebio; 1 min), differentiated (1% HCl in alcohol; 10–20 s), blued under running water (5 min), and counterstained with eosin (Servicebio; 2 min). Masson’s trichrome staining followed sequential treatment with iron hematoxylin (7.5 min), acid fuchsin (65 °C; 6 min), phosphomolybdic acid (1%; 2 min), and aniline blue (Servicebio; 5 min), with a final rinse in 1% acetic acid. All sections were dehydrated, cleared, mounted, and scanned using a digital slide scanner (Aperio AT2, Leica).

### 2.17. Cell Viability Assay

DPCs were seeded at 5 × 10^3^ cells/well in 96-well plates. After overnight adhesion, cells were treated with varying concentrations of Mdivi-1 (0, 10^−4^, 10^−5^, 10^−6^, 10^−7^, 10^−8^ mol/L) for 0, 1, 3, 5, and 7 days. Cell viability was assessed by adding CCK-8 reagent (Biofiven, Guangzhou, China) and measuring the absorbance at 450 nm after 2 h incubation. Data were normalized to untreated controls. Based on cell viability assay and functional experiment (Figure S1), 10^−6^ mol/L Mdivi-1 was selected as the working concentration for subsequent experiments.

### 2.18. Tooth Germs Culture

Mdivi-1 (10 mM stock solution in DMSO, MCE, Monmouth Junction, NJ, USA) was diluted to a working concentration of 10^−6^ mol/L in culture medium. The final DMSO concentration in the working solution was 0.01%. Control groups received no treatment.

Mandibular first molar tooth germs were aseptically dissected from E19.5 SD rat embryos under a stereomicroscope (Carl Zeiss AG). Organ culture was performed according to the protocol of a study [23]. Briefly, each tooth germ was placed on a nitrocellulose membrane (0.4 µm pore size; EMD Millipore) supported by a stainless steel grid in a 12-well plate. The culture medium consisted of α-MEM supplemented with 10% fetal bovine serum, 100 U/mL penicillin, 100 µg/mL streptomycin, and 2 mM glutamine. For differentiation, the medium was further supplemented with OS medium components as described in Section 2.2, with or without 10^−6^ mol/L Mdivi-1. The cultures were maintained at 37 °C in a humidified atmosphere of 5% CO_2_. Following a 1-day adherence period, the tooth germs were treated for 14 days, and the medium was changed every 2 days. Tooth germ morphology was observed under a microscope, and areas were measured using ImageJ software. The cultured tooth germs were then fixed, embedded, sectioned, and subjected to H&E staining.

### 2.19. Image Analysis

All image quantifications were performed using ImageJ 1.54g software.

For confocal microscopy, mitochondrial morphology parameters (aspect ratio, form factor, perimeter, and area) were analyzed using the “Mitochondrial Analyzer” plugin, and co-localization was quantified using Pearson’s correlation coefficient. At least 10 cells per condition were analyzed from 5–10 random fields.

For TEM analysis, ER–mitochondria distance was measured using the “Measure” tool, and MAMs were identified as regions with a distance ≤ 30 nm. Parameters quantified included MAMs/mitochondria perimeter ratio, ER–mitochondria distance, and ER–mitochondria contact coefficient (ERMICC). At least 10 mitochondria per condition were analyzed from 15–20 random TEM fields, with fields selected systematically to avoid bias.

All quantifications were performed blinded to experimental group assignment.

### 2.20. Statistical Analysis

All experiments were performed with at least three biological replicates. Technical replicates were averaged before statistical analysis. Data are presented as mean ± standard deviation (SD) of biological replicates. Statistical analyses were performed using GraphPad Prism 8.0 (GraphPad Software, San Diego, CA, USA) with appropriate parametric or non-parametric tests. Fixed-effects models were used for all comparisons. Normality was assessed using the Shapiro–Wilk test, and homogeneity of variances was verified by Levene’s test. For two-group comparisons, an unpaired Student’s *t*-test (parametric) was used. Multiple groups were compared using one-way ANOVA with Tukey’s post hoc test (parametric). A *p*-value of < 0.05 was considered statistically significant.

## 3. Results

### 3.1. Early Mitochondrial Fission and Increased Mitochondria–ER Contacts Facilitate Odontogenic Differentiation of DPCs

**Figure 1:**
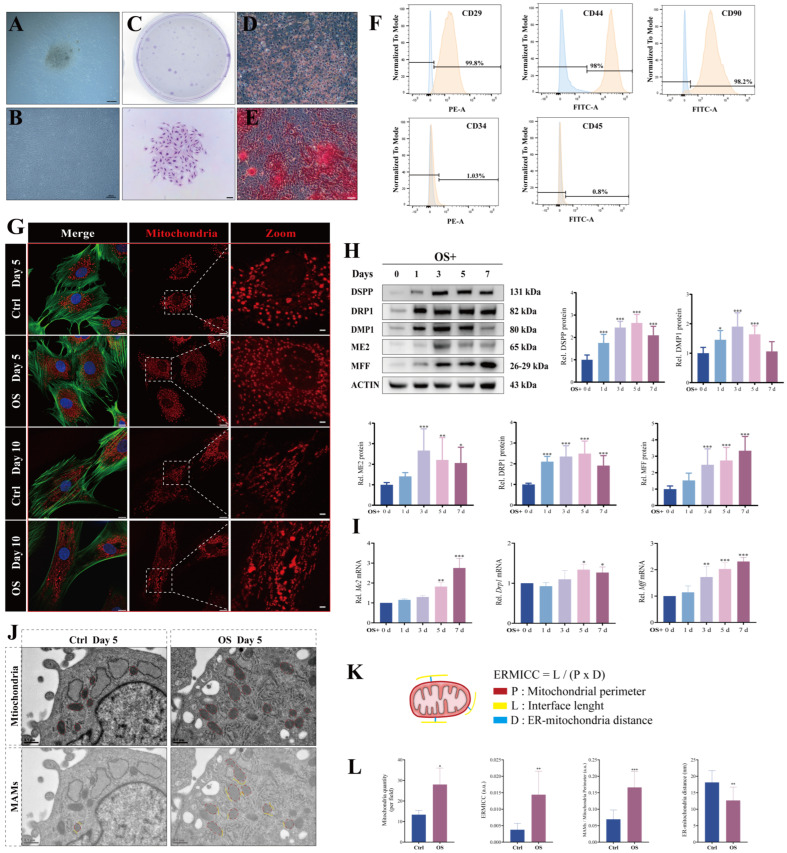
Identification of DPC stemness and patterns of mitochondrial fission and MAMs during odontogenic differentiation. (**A**,**B**) Primary culture of DPCs on day 3 (**A**) and at passage 3 (**B**). Scale bar: 200 µm. (**C**) Macroscopic (top) and microscopic (bottom) images of the colony-forming unit. Scale bar: 200 µm. (**D**) Oil Red O staining of lipid droplets after adipogenic induction, and (**E**) ARS of mineralized nodules after osteo/odontogenic induction. Scale bar: 50 µm. (**F**) Flow cytometric analysis of MSC markers (CD29, CD44, CD90) and hematopoietic markers (CD34, CD45). (**G**) Fluorescence images showing mitochondria (red), F-actin (green), and DAPI-stained nuclei (blue) on days 5 and 10. DPCs were treated with basal growth medium (Ctrl) and osteogenic/odontogenic medium (OS), respectively. Scale bar: 10 µm (left two panels) and 2 µm (right panel). (**H**) Western blot images and relative quantification of indicated protein levels in OS treated DPCs (days 0–7). *n* = 9 independent biological replicates. (**I**) Relative mRNA levels of key markers in OS treated DPCs (days 0–7). *n* = 6 independent biological replicates. (**J**) TEM images of DPCs with or without OS treatment for 5 days. Mitochondria and ER are pseudo-colored in red and yellow, respectively, highlighting the mitochondria and MAMs structure. Scale bar: 0.5 µm. (**K**) Schematic of the ER–mitochondria contact coefficient (ERMICC). (**L**) Quantification of mitochondrial number, ERMICC, MAMs/mitochondria perimeter ratio and mitochondria–ER distance from TEM analysis in (**J**). *n* = 3 independent biological replicates. Data are presented as mean ± SD. Compared with respective controls. * *p* < 0.05, ** *p* < 0.01, *** *p* < 0.001. Original images of (**H**) can be found in Supplementary Materials.

Primary DPCs were successfully isolated from rat molar germs using the explant method (Figure 1A). After expansion to the third passage (P3), the cells displayed a typical fibroblast-like morphology (Figure 1B) and formed numerous colonies, indicative of robust proliferative potential (Figure 1C). The DPCs also demonstrated multi-lineage differentiation capacity, as evidenced by the formation of lipid droplets (adipogenic, Figure 1D) and mineralized nodules (osteo/odontogenic, Figure 1E). Phenotypic analysis by flow cytometry confirmed these cells were positive for MSC markers (CD29, CD44, and CD90) and negative for hematopoietic markers (CD34 and CD45) (Figure 1F). Together, these findings confirm that the isolated DPCs possess key MSC characteristics.

To investigate the role of mitochondrial fission and MAMs during the odontogenic differentiation, we first examined mitochondrial morphology. IF analysis revealed dynamic morphological changes in mitochondria throughout the differentiation process (Figure 1G). At the early stage (day 5), mitochondria appeared smaller and more numerous, suggesting increased fission activity. By the later stage (day 10), a pronounced elongation was observed, indicative of enhanced mitochondrial biogenesis and fusion, which was consistent with our previous findings [19,24]. At the molecular level, Western blot showed that the expression levels of ME2, DRP1, MFF, DSPP, and DMP1 were progressively upregulated over 7 days of mineralization induction, with all increases reaching statistical significance by day 5 (Figure 1H). A consistent trend was observed for the mRNA expression of *Drp1* and *Mff* (Figure 1I). These findings indicate that mitochondrial fission is activated during the early phase of odontogenic differentiation in DPCs.

We next examined ER–mitochondria interactions. TEM was used to detect the changes as the gold standard for visualizing MAM ultrastructure [25]. In the OS-treated group, there was an increase in mitochondrial number, a higher proportion of MAMs, and a reduced distance between the ER and mitochondria (Figure 1J,L). We also applied the ER–mitochondria contact coefficient (ERMICC), a quantitative metric that accounts for interaction length and mitochondrial perimeter, thereby normalizing for variations in mitochondrial size [22] (Figure 1K). ERMICC also showed a significant elevation in OS-treated DPCs. Together, these results suggest that enhanced MAM assembly may play a functional role in this process.

### 3.2. DRP1 Regulates Odontogenic Differentiation by Coordinating Mitochondrial Fission and MAM Formation

**Figure 2:**
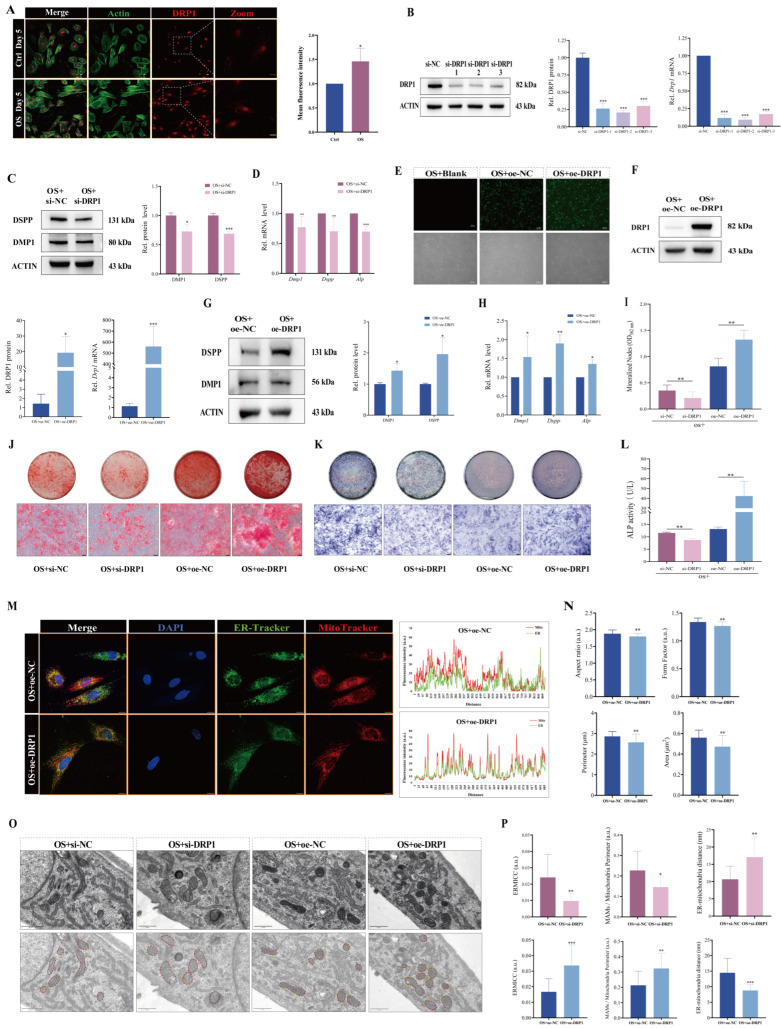
DRP1-mediated mitochondrial fission and MAMs are essential for odontogenic differentiation. (**A**) IF staining for DRP1 protein in DPCs cultured with or without OS treatment for 5 days and its semi-quantitative analysis. *n* = 3 independent biological replicates. Scale bar: 10 µm. (**B**) Knockdown efficiency of si-DRP1 constructs assessed by Western blot and RT-qPCR. *n* = 4 independent biological replicates. (**C**) Protein and (**D**) mRNA levels of key differentiation markers after DRP1 knockdown. *n* = 3 independent biological replicates. (**E**) DPCs transduced with GFP-tagged DRP1 overexpressing lentivirus. Scale bar: 200 µm. (**F**) Overexpression efficiency of oe-DRP1 lentivirus analyzed by Western blot and RT-qPCR. *n* = 3 independent biological replicates. (**G**) Protein and (**H**) mRNA levels of key differentiation markers after DRP1 overexpression. *n* = 3 independent biological replicates. (**I**) Semi-quantitative analysis of mineralized nodules shown in (**J**). (**J**) Macroscopic (top) and microscopic (bottom) images of ARS staining in DPCs transfected with indicated constructs and cultured in OS medium. Scale bar: 200 µm. *n* = 4 independent biological replicates. (**K**) ALP staining and (**L**) ALP activity assay cultured in control and DRP1 modulated DPCs. *n* = 3 independent biological replicates. Scale bar: 200 µm. (**M**) Representative confocal images of oe-NC and oe-DRP1 DPCs stained with MitoTracker (red) and ER-Tracker (green), with co-localization line profile. Scale bar: 10 µm. Red lines indicate the positions where fluorescence intensity co-localization analysis was performed. (**N**) Quantitative analysis of mitochondrial morphology parameters (aspect ratio, form factor ratio, perimeter, and area) from (**M**). *n* = 22 independent biological replicates. (**O**) TEM images of mitochondria–ER contacts in different treatment groups. Mitochondria and ER are pseudo-colored in red and yellow, respectively, highlighting the MAMs structure. Scale bar: 0.5 µm. (**P**) Quantification of MAM frequency from TEM images in (**O**). *n* = 4 independent biological replicates. Data are presented as mean ± SD. Compared with respective controls. * *p* < 0.05, ** *p* < 0.01, *** *p* < 0.001. Original images of (**B**,**C**,**F**,**G**) can be found in Supplementary Materials.

To investigate the role of DRP1 in odontogenic differentiation, we first confirmed that its expression was upregulated upon OS induction (Figure 2A). We then employed siRNA-mediated knockdown and validated that all three si-DRP1 constructs effectively suppressed DRP1 expression. The construct si-DRP1-2, which showed the strongest knockdown efficiency (Figure 2B), was selected for all subsequent experiments (hereafter referred to as si-DRP1). DRP1 knockdown significantly impaired odontogenic differentiation, as shown by the downregulation of DMP1 and DSPP proteins (Figure 2C) and the reduction in *Dmp1*, *Dspp*, and *Alp* mRNA levels (Figure 2D). Subsequently, GFP-tagged lentivirus was used to overexpress DRP1 in DPCs. Microscopic observation revealed that the fluorescence positivity rates in both the negative control (oe-NC) group and the overexpression (oe-DRP1) group exceeded 80% compared with uninfected cells (Blank), indicating successful infection (Figure 2E). Successful transfection was further confirmed by both protein and mRNA expression levels of DRP1 (Figure 2F). In contrast, DRP1 overexpression enhanced the expression of these markers at both protein (Figure 2G) and mRNA (Figure 2H) levels.

Functionally, DRP1 knockdown strongly inhibited mineralized nodule formation and reduced ALP activity, while its overexpression markedly promoted both processes (Figure 2I–L). We further explored the underlying mechanism by examining its impact on mitochondrial fission and inter-organelle communication. Confocal microscopy, widely used to study mitochondria–ER interactions [26], showed that DRP1 overexpression concurrently enhanced mitochondria–ER association and induced mitochondrial fragmentation (Figure 2M). This was evidenced by a marked reduction in key parameters, including aspect and form factor ratio (reflecting morphology of mitochondria), as well as perimeter and area (indicating size of mitochondria) (Figure 2N), consistent with enhanced fission activity. Moreover, TEM revealed that DRP1 expression levels positively correlated with MAM frequency (Figure 2O,P). Together, these results demonstrate that DRP1 is a critical regulator of odontogenic differentiation, likely through coordinating mitochondrial fission and MAM formation.

### 3.3. DRP1-Mediated Mitochondria Fission Is Required for Odontoblast Differentiation and Tooth Development

**Figure 3:**
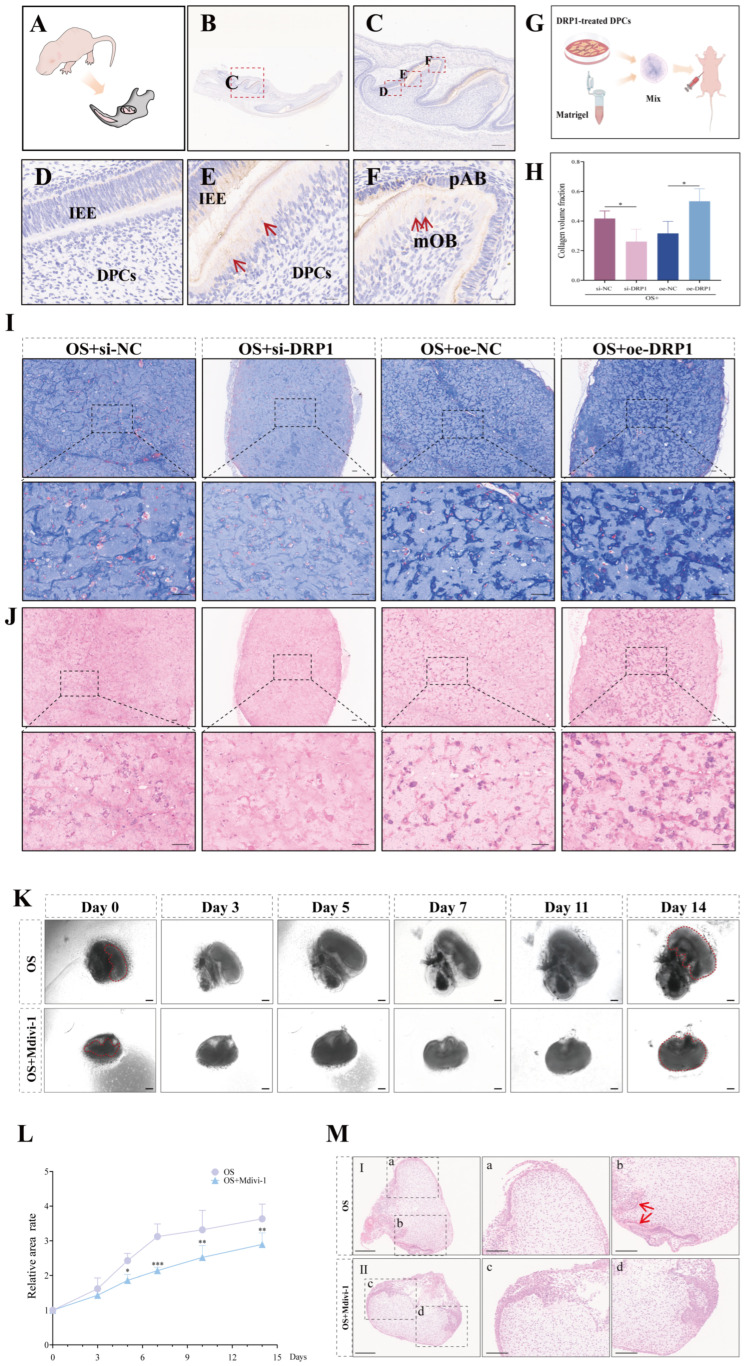
The role of DRP1 in odontogenic differentiation and dental tissue formation. (**A**) Schematic diagram of the mandible from a PN3–5 SD rat. (**B**,**C**) IHC of DRP1 in the first molar germ. Boxed regions in top panels are magnified below. Scale bar: 400 µm. (**D**–**F**) Gradual increase in DRP1 expression during natural odontoblast differentiation. Red arrows indicate DRP1-positive cells. Scale bar: 100 µm. (**G**) Schematic diagram of the in vivo transplantation experiment. (**H**) Quantitative analysis of collagen volume fraction. (**I**) Masson’s trichrome and (**J**) H&E staining of collagen matrix formation in vivo. *n* = 4 independent biological replicates. Scale bars: 400 µm (upper panels), 100 µm (lower panels). (**K**) Ex vivo culture of mandibular first molar germs from E19.5 rats treated with OS or OS + Mdivi-1 for 14 days. Scale bar: 200 µm. (**L**) The relative growth rate of tooth germ area was analyzed at indicated time points. One tooth germ sample in the OS group was integrally excluded from statistical analysis due to microbial contamination during long-term culture. Valid sample size: OS group, *n* = 4; OS + Mdivi-1 group, *n* = 5. (**M**) H&E staining of tooth germ culture with OS or OS + Mdivi-1 for 14 days. Boxed regions in left panels are shown at higher magnification in the right panels. Scale bar: 200 µm (**I**,**II**), 100 µm (**a**–**d**). Red arrows indicate predentin. DPCs, dental papilla cells; IEE, inner enamel epithelium; pAB, preameloblast; mOB, mature odontoblast. Data are presented as mean ± SD. Compared with respective controls. * *p* < 0.05, ** *p* < 0.01, *** *p* < 0.001.

We first investigated the spatiotemporal expression of DRP1 during odontogenesis. IHC analysis of PN 3–5 rat molar germs showed that DRP1 expression increased during odontoblast differentiation and maturation (Figure 3A–F), suggesting its involvement in this process.

To functionally validate this developmental observation, we performed an in vivo transplantation assay (Figure 3G). Histological analysis demonstrated that DRP1 knockdown significantly impaired collagen matrix formation, as evidenced by a reduced collagen volume fraction (Figure 3H) and attenuated staining in both Masson’s trichrome (Figure 3I) and H&E (Figure 3J) sections. Conversely, DRP1 overexpression enhanced collagen deposition (Figure 3H–J).

We further confirmed these findings using an ex vivo tooth germ culture system. The compound Mdivi-1 has been identified as a mitochondrial fission inhibitor that targets the evolutionarily conserved GTPase DRP1 [27]. We first determined its optimal concentration through cell proliferation and mineralization assays (Figure S1). A concentration of 10^−6^ mol/L Mdivi-1 was found to inhibit the mineralization of DPCs without exerting cytotoxic effects on cell proliferation, whereas other concentrations showed no promotive activity on mineralization. Accordingly, treatment with 10^−6^ mol/L Mdivi-1 significantly suppressed the growth of molar germs compared with the OS-treated group from day 5 (Figure 3K,L). To account for variations in area caused by differences in germ attachment orientation, the relative area growth rate was used for quantification. After 14 days of culture, H&E staining revealed that, while both the OS and OS + Mdivi-1 groups exhibited columnar morphology and nuclear polarization of odontoblasts, predentin formation was observed only in the OS group (Figure 3M, red arrow). These findings indicate that DRP1-mediated mitochondrial fission plays a crucial role in tooth germ development.

Collectively, these results from developmental expression analyses, in vivo functional assays, and ex vivo organ cultures demonstrate that DRP1 plays an essential role in regulating odontogenic differentiation and tooth development.

### 3.4. Molecular Modulation of ME2 Reveals Its Essential Role in Odontogenic Differentiation via Regulating Mitochondrial Fission and MAMs

**Figure 4:**
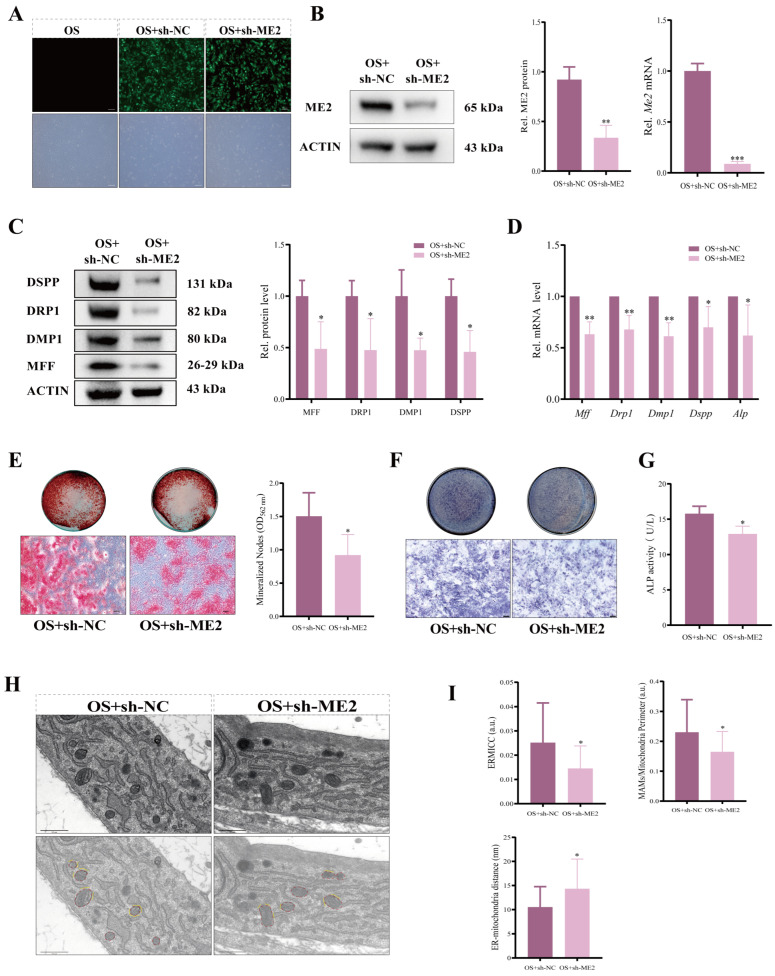
Knockdown of ME2 inhibits the odontogenic differentiation of DPCs and reduces MAM formation. (**A**) DPCs transduced with GFP-tagged lentivirus for ME2 knockdown exhibited high fluorescence positivity rates. Scale bar: 200 µm. (**B**) Validation of ME2 knockdown efficiency at protein and mRNA levels. *n* = 3 independent biological replicates. (**C**) Protein and (**D**) mRNA levels of indicated markers in ME2-knockdown DPCs. *n* = 3 independent biological replicates. (**E**) ARS and semi-quantitative analysis of mineralized nodule formation. *n* = 6 independent biological replicates. Scale bar: 200 µm. (**F**) ALP staining and (**G**) ALP activity assay in ME2-knockdown DPCs. *n* = 3 independent biological replicates. Scale bar: 200 µm. (**H**) TEM images of mitochondria–ER contacts. Mitochondria and ER are pseudo-colored in red and yellow, respectively, highlighting the MAMs structure. Scale bar: 0.5 µm. (**I**) Quantification of MAM frequency from TEM images in (**H**). *n* = 4 independent biological replicates. Data are presented as mean ± SD. Compared with respective controls. * *p* < 0.05, ** *p* < 0.01, *** *p* < 0.001. Original images of (**B**,**C**) can be found in Supplementary Materials.

**Figure 5:**
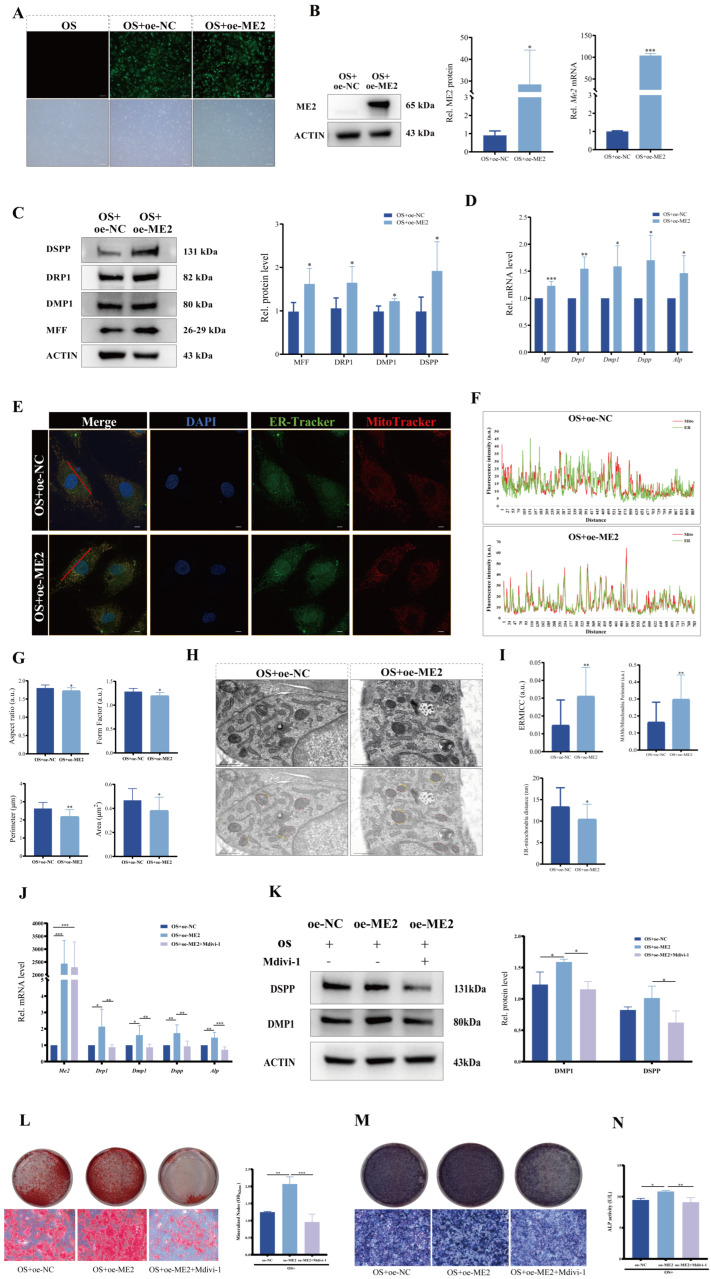
ME2 overexpression promotes odontogenic differentiation by regulating DRP1-mediated mitochondrial fission. (**A**) DPCs transduced with GFP-tagged lentivirus for ME2 overexpression exhibited high fluorescence positivity rates. Scale bar: 200 µm. (**B**) Validation of ME2 overexpression by Western blot and RT-qPCR. *n* = 3 independent biological replicates. (**C**) Protein (*n* = 4) and (**D**) mRNA (*n* = 5) levels of indicated markers in ME2 overexpressing DPCs. Protein (*n* = 4) and mRNA (*n* = 5), independent biological replicates. (**E**) Representative confocal images of oe-NC and oe-ME2 DPCs stained with MitoTracker (red) and ER-Tracker (green). Red lines indicate the positions where fluorescence intensity co-localization analysis was performed. Scale bar: 10 µm. (**F**) Co-localization line profile analysis from (**E**). (**G**) Quantitative analysis of mitochondrial morphology parameters from (**E**). *n* = 3 independent biological replicates. (**H**) TEM images of mitochondria–ER contacts. Mitochondria and ER are pseudo-colored in red and yellow, respectively, highlighting the MAMs structure. Scale bar: 0.5 µm. (**I**) Quantification of MAM frequency from TEM images in (**H**). *n* = 4 independent biological replicates. (**J**) mRNA expression levels of *Me2*, *Drp1*, *Dmp1*, *Dspp*, and *Alp* in DPCs in oe-NC, oe-ME2, and oe-ME2 + Mdivi-1 groups after 5 days of mineralization induction. *n* = 3 independent biological replicates. (**K**) Protein expression levels of DMP1 and DSPP in DPCs in oe-NC, oe-ME2, and oe-ME2 + Mdivi-1 groups after 5 days of mineralization induction, along with semi-quantitative analysis. *n* = 3 independent biological replicates. (**L**) ARS staining and semi-quantitative analysis of mineralized nodule formation. *n* = 3 independent biological replicates. Scale bar: 200 µm. (**M**) ALP staining and (**N**) ALP activity assay. Scale bar: 200 µm. *n* = 3 independent biological replicates. Data are presented as mean ± SD. Compared with respective controls. * *p* < 0.05, ** *p* < 0.01, *** *p* < 0.001. Original images of (**B**,**C**,**K**) can be found in Supplementary Materials.

To elucidate the functional role of ME2 in odontogenic differentiation, we performed both loss-of-function and gain-of-function studies in DPCs. Lentiviral infection was used to achieve knockdown (sh-ME2) and overexpression (oe-ME2) of ME2. Fluorescence microscopy revealed that the positivity rates in both groups exceeded 80% (Figure 4A and Figure 5A), and the efficiency of both knockdown and overexpression was subsequently validated at both the mRNA and protein levels (Figure 4B and Figure 5B). ME2 deficiency markedly impaired odontogenic differentiation, as evidenced by the downregulation of key regulators MFF, DRP1 and differentiation markers DMP1, DSPP, and ALP at both protein and mRNA levels (Figure 4C,D). Functionally, this resulted in reduced mineralized nodule formation and decreased ALP expression and activity (Figure 4E–G). Conversely, overexpression of ME2 significantly enhanced the odontogenic program, upregulating the same set of markers (Figure 5C,D).

We next investigated whether these phenotypic changes were associated with alterations in mitochondrial morphology and mitochondria–ER contact. TEM revealed that ME2 knockdown disrupted mitochondria–ER architecture, leading to a decreased ERMICC, increased inter-organelle distance, and a reduced MAMs/mitochondria perimeter ratio (Figure 4H,I). In contrast, ME2 overexpression strengthened mitochondria–ER connectivity, increasing the ERMICC and reducing the inter-organelle distance (Figure 5H,I). Consistent with these ultrastructural findings, confocal microscopy and morphometric analysis confirmed that ME2 overexpression enhanced mitochondria–ER association and promoted mitochondrial fragmentation. These changes were evidenced by an increase in colocalization and concurrent decreases in key mitochondrial morphometric parameters (Figure 5E–G).

To determine whether the pro-differentiation effect of ME2 is mediated through DRP1-dependent mitochondrial fission, rescue experiments were performed using the DRP1 inhibitor Mdivi-1. Following 5 days of mineralization induction, the upregulation of odontogenic markers (DMP1, DSPP, and ALP) induced by ME2 overexpression was markedly attenuated by co-treatment with Mdivi-1 at both the mRNA (Figure 5J) and protein (Figure 5K) levels. Consistently, ARS staining (Figure 5L), ALP staining (Figure 5M), and ALP activity assays (Figure 5N) demonstrated that the enhanced mineralized nodule formation and ALP activity in ME2-overexpressing cells were significantly suppressed by Mdivi-1 treatment.

Collectively, these complementary findings establish ME2 as a critical regulator of odontogenic differentiation, acting through the modulation of DRP1-mediated mitochondrial fission and the maintenance of mitochondria–ER contact integrity. ME2 thereby serves as a key coordinator of metabolic and structural remodeling during odontoblast differentiation.

## 4. Discussion

The differentiation of DPCs into functional odontoblasts is a critical process in dentinogenesis. While metabolic reprogramming is known to accompany odontogenic differentiation [28,29], the specific mechanisms underlying the balance of mitosis and fusion in mitochondrial dynamics and the organelle-mediated communication between mitochondria and ER remain incompletely understood. This study elucidates a coordinated program of mitochondrial fission and MAM formation that is essential for odontogenic differentiation and identifies ME2 and DRP1 as its central regulators.

Mitochondrial fission, the counter-process to fusion, is equally critical for mitochondrial quality control, metabolic adaptation, and cellular homeostasis [30]. Consequently, its absence results in elongated mitochondrial networks and ultimately, compromised cellular function and viability [31,32,33,34]. Homeostasis of mitochondrial quantity and morphology requires a precise balance between fusion and fission [35]. Our study found that odontogenic differentiation in DPCs is accompanied by an overall increase in mitochondrial abundance, and the expression of fission-related proteins DRP1 and MFF. This pattern suggests that mitochondrial fission is an actively regulated initiating event in the differentiation process, consistent with observations in other stem cell systems, where a transient fission burst facilitates metabolic reprogramming and suppresses self-renewal [36,37]. Such activation may help meet the metabolic and signaling demands as cells shift from stemness maintenance to a differentiation-prone state [38,39]. However, in inflammatory aging [13] or high glucose [14] model of dental pulp cells, DRP1 upregulation is associated with reduced cell proliferation and differentiation capacity, as well as elevated levels of senescence and apoptosis. The physiological significance of mitochondrial fission may be context-dependent: physiological, timely fission promotes stem cell differentiation and tissue regeneration, whereas pathological, sustained fission may lead to mitochondrial fragmentation, dysfunction, and cellular senescence. Consistent with our observation, mitochondrial fission has been shown promoting differentiation of apical tooth papilla cells [40] and osteogenesis [41]. The absence of inflammatory or senescent stimuli aligns more closely with physiological regulatory characteristics, suggesting a positive driving effect of mitochondrial fission on dentin formation.

DRP1, as the master executor of mitochondrial fission, is recruited to the mitochondrial membrane primarily by the receptor protein MFF during normal cell growth and differentiation in mammalian cells [17,42]. Our study demonstrates that DRP1-mediated mitochondrial fission serves as a key driver of odontogenic differentiation in DPCs, supported by both in vitro and in vivo evidence. Furthermore, treatment with the mitochondrial fission inhibitor Mdivi-1 impaired tooth germ development, providing strong evidence for the physiological relevance of this mechanism. These findings align with previous reports showing that DRP1-mediated mitochondrial fission is essential for normal embryonic development and serves as an early key event in stem cell differentiation. DRP1 knockout in mice leads to developmental defects extending across multiple tissues and organs, including the heart, liver, and neural tube, ultimately resulting in embryonic lethality [11,43,44]. DRP1-mediated mitochondrial fission has also been identified as a key driver of chondrogenic [10] and adipogenic [45] differentiation of MSCs, as well as the activation and regeneration of neural [46] and muscle stem cells [47]. These observations suggest that DRP1-mediated mitochondrial fission may represent a common regulatory mechanism underlying stem cell differentiation. Our study further extends this concept to the odontoblastic differentiation of DPCs, thereby enriching the mitochondrial regulatory network governing stem cell differentiation.

Our data further reveal that the mitochondrial fission in DPCs is tightly coupled with enhanced MAM formation, suggesting a functional linkage crucial for odontoblastic differentiation. This finding aligns with the established role of MAMs as a conserved platform for mitochondrial fission, where ER tubules localize to fission sites to induce local constriction, enabling DRP1 oligomer assembly and final scission [48]. MFF-mediated recruitment of DRP1 at MAMs supports midzone fission required for normal cell growth and division [49,50]. However, multiple earlier studies on MAMs-associated fission have been confined to housekeeping functions in immortalized cell lines, potentially leaving the regulatory mechanisms under DPC differentiation less explored. Importantly, the functional dependence on MAMs varies across differentiation lineages. For instance, increased MAMs during cardiomyocyte differentiation ensure efficient Ca^2+^ transfer from the ER into mitochondria [51], and neuronal differentiation demands exceptionally high precision in calcium signaling [52]. In contrast, adipogenic differentiation places greater demands on MAM-mediated lipid metabolism and the expansion of secretory functions [53]. In progenitor cells of mineralized tissues such as skeletal [54] and dental lineages [18], differentiation involves enhanced secretory activity and increased extracellular matrix production. These processes require extensive ER expansion and mitochondrial support. Thus, the specific MAM-dependent mechanism engaged may vary depending on the metabolic and secretory demands of the target lineage.

Here, we provide the first evidence that ME2 critically regulates mitochondrial fission and MAM integrity by targeting DRP1 and MFF as downstream effectors, as demonstrated by loss- and gain-of-function assays. This novel regulatory function extends beyond well-established roles of ME2. Traditionally, ME2 catalyzes the oxidative decarboxylation of malate to pyruvate and generates NADPH to fuel reductive biosynthetic pathways [48]. Through its metabolic role, ME2 regulates stem cell differentiation. During osteogenic differentiation of MSCs, ME2 is upregulated and promotes differentiation by generating NADPH to maintain redox balance [55] while paradoxically correlating with less differentiated states in cancer stem cells [56]. Additionally, recent studies have uncovered non-metabolic functions of ME2, including upregulation of OPA1 and MFN2 to promote mitochondrial fusion [19] and suppression of PINK1-Parkin-mediated mitophagy via TRIM25 binding [57]. None of these prior studies linked ME2 to mitochondrial fission or MAMs. Our findings therefore address this gap in the literature, suggesting that ME2 integrates metabolism, fusion, mitophagy, and fission, thereby substantially expanding the understanding of its regulatory network and supporting its potential as a therapeutic target for pulp regeneration.

Rescue experiments further confirmed that ME2 requires DRP1 to drive odontogenic differentiation, placing DRP1-dependent fission downstream of ME2. On the one hand, further investigation is needed to establish whether DRP1 functions as a direct downstream target of ME2. On the other hand, we propose a hypothetical mechanism whereby ME2-derived NADPH may fuel phospholipid synthesis at MAMs, where the requisite enzymes for phospholipid biosynthesis reside [58]. Lipids such as phosphatidylethanolamine (PE) and cardiolipin (CL) [59] are fundamental for mitochondrial and ER membranes [60] and are essential for DRP1 oligomerization and GTPase activity [61]. Thus, ME2-derived NADPH could create a lipid microenvironment permissive for DRP1 activation at MAMs, positioning ME2 activity as a metabolic prerequisite for initiating the fission-differentiation program. However, this model remains speculative and requires direct visualization of NADPH and lipid dynamics at MAMs to definitively establish spatial coupling with DRP1 activation. Additionally, ME2-generated pyruvate enters the tricarboxylic acid cycle to fuel oxidative phosphorylation, influencing ATP/AMP ratios and potentially activating AMPK, which is known to promote DRP1 recruitment by phosphorylating MFF [62]. These alternative pathways, while speculative, offer additional mechanistic possibilities that warrant future investigation.

### Limitation

However, several limitations should be noted. First, while we demonstrate that ME2 promotes DRP1 recruitment and MAM assembly, the precise molecular mechanism, particularly whether DRP1 is a direct downstream target of ME2, requires further investigation. Second, the observed correlation between mitochondrial fission and MAM formation awaits causal validation using more selective tools, such as MAM-specific perturbations. Third, the ex vivo tooth germ culture model does not fully recapitulate the native microenvironment and potentially omits paracrine and mechanical influences. Fourth, although Mdivi-1 is widely used as a pharmacological inhibitor of DRP1, its off-target effects on mitochondrial complex I and ion channels require caution [63]. Complementary approaches, such as conditional genetic knockdown or knockout of DRP1, will be essential to confirm its specific role in odontogenic differentiation. Addressing these aspects in future studies will further strengthen the translational relevance of our work.

## 5. Conclusions

In summary, our study defines a previously unrecognized ME2-DRP1-MAM axis that is essential for odontoblast differentiation and dentin formation. We establish through in vitro and in vivo experiments that mitochondrial fission and MAM formation are spatiotemporally coupled during this process, with ME2 acting upstream to promote MAM integrity, thereby facilitating DRP1-dependent fission. This axis integrates metabolic signaling with mitochondrial dynamics to drive lineage commitment, providing a rationale for developing small-molecule modulators or bioactive scaffolds that precisely tune this axis, shifting dental tissue engineering strategies from passive support toward active control of progenitor cell metabolism and organelle dynamics to achieve more predictable regeneration.

## Figures and Tables

**Table 1:** Antibodies.

Antibodies	Dilution	Purchased from
anti-DSPP primary antibody	1:500 for WB	Santa Cruz, Santa Cruz, CA, USA
anti-DMP1 primary antibody	1:1000 for WB	Affinity, Lexington, MA, USA
anti-ACTIN primary antibody	1:3000 for WB	Affinity, Lexington, MA, USA
anti-ME2 primary antibody	1:1000for WB	Abcam, Cambridge, UK
anti-DRP1 primary antibody	1:1000 for WB 1:200 for IF 1:100 for IHC	Affinity, Lexington, MA, USA
anti-MFF primary antibody	1:1000 for WB	Affinity, Lexington, MA, USA
Actin-Tracker Green-488	1:100	Beyotime, Shanghai, China
Dylight 488/594 conjugated secondary antibody	1:200	EarthOx, Burlingame, CA, USA
HRP-conjugated secondary antibodies	1:1000	Beyotime, Shanghai, China

**Table 2:** RT-qPCR primers.

Gene	Forward Primer (5′-3′)	Reverse Primer (5′-3′)	RefSeq ID
*Dspp*	ACAGCGACAGCGACGATTC	CCTCCTACGGCTATCGACTC	NM_012790.3
*Dmp1*	ACCAAAATACTGAATCTGAAAGCTC	TGCTGTCCGTGTGGTCACTA	NM_203493.5
*Alp*	GGAAGGAGGCAGGATTGA	TCAGCAGTAACCACAGTCA	NM_013059.3
*Me2*	ACTTACAGAGGGCAGGTGCTTG	TCGGGCTTCACAGAGAATAACAG	NM_063277298.1
*Mff*	ACATGCGCATTGGAGCAGTA	GCCCCACTCACCAAATGAGA	NM_032901476.1
*Drp1*	TGACATCTTGACCGCCATTA	TGGGCTCCTCTAGACGCTTA	NM_032899813.1
*β-actin*	CGGTCAGGTCATCACTATC	CAGCACTGTGTTGGCATA	NM_031144.3

**Table 3:** siRNA sequences of DRP1.

Gene	Sense (5′-3′)	Antisense (5′-3′)
*si-NC*	UUCUCCGAACGUGUCACGUTT	ACGUGACACGUUCGGAGAATT
*si-DRP1-1*	GGUGCUAGGAUUUGUUAUATT	UAUAACAAAUCCUAGCACCTT
*si-DRP1-2*	GGGCUAAUGAACAAUAACATT	UGUUAUUGUUCAUUAGCCCTT
*si-DRP1-3*	GCAGAACUCUAGCUGUAAUTT	AUUACAGCUAGAGUUCUGCTT

## Data Availability

The original contributions presented in this study are included in the article/Supplementary Materials. Further inquiries can be directed to the corresponding authors.

## Supplementary Materials

The following supporting information can be downloaded at: https://www.mdpi.com/article/10.3390/biom16050664/s1, Figure S1: Evaluation of Mdivi-1 effects on DPCs. Original images of Figure 1H, Figure 2B,C,F,G, Figure 4B,C and Figure 5B,C,K can be found in https://doi.org/10.5281/zenodo.19634238 access date: 23 March 2026.

## Abbreviations

The following abbreviations are used in this manuscript:

DPCs	dental papilla cells
MAMs	mitochondria-associated endoplasmic reticulum membranes
DRP1	dynamin-related protein 1
ME2	malic enzyme 2
MFF	mitochondrial fission factor
ER	endoplasmic reticulum
OPA1	optic atrophy 1
MFN2	mitofusin 2
SD	Sprague-Dawley
OS	osteogenic/odontogenic medium
ARS	alizarin red staining
MSC	mesenchymal stem cell
RT-qPCR	real-time quantitative polymerase chain reaction
TEM	transmission electron microscopy
IF	immunofluorescence
DSPP	dentin sialophosphoprotein
DMP1	dentin matrix protein 1
ACTIN	β-actin
ALP	alkaline phosphatase
IHC	immunohistochemistry
H&E	hematoxylin and eosin
ERMICC	ER–mitochondria contact coefficient
FIS1	mitochondrial fission protein 1
PE	phosphatidylethanolamine
CL	cardiolipin
