# Supplementary material for: Malic Enzyme 2 Regulates Dynamin-Related Protein 1-Dependent Mitochondrial Fission and Mitochondria-Associated Membranes to Drive Odontogenic Differentiation: An In Vitro and In Vivo Study
Source: Biomolecules. 2026 Apr 30;16(5):664. doi: 10.3390/biom16050664 (PMC13204223; doi:10.3390/biom16050664)
Supplement: Supplementary file 1 [file biomolecules-16-00664-s001.zip › biomolecules-4248036-supplementary.pdf]

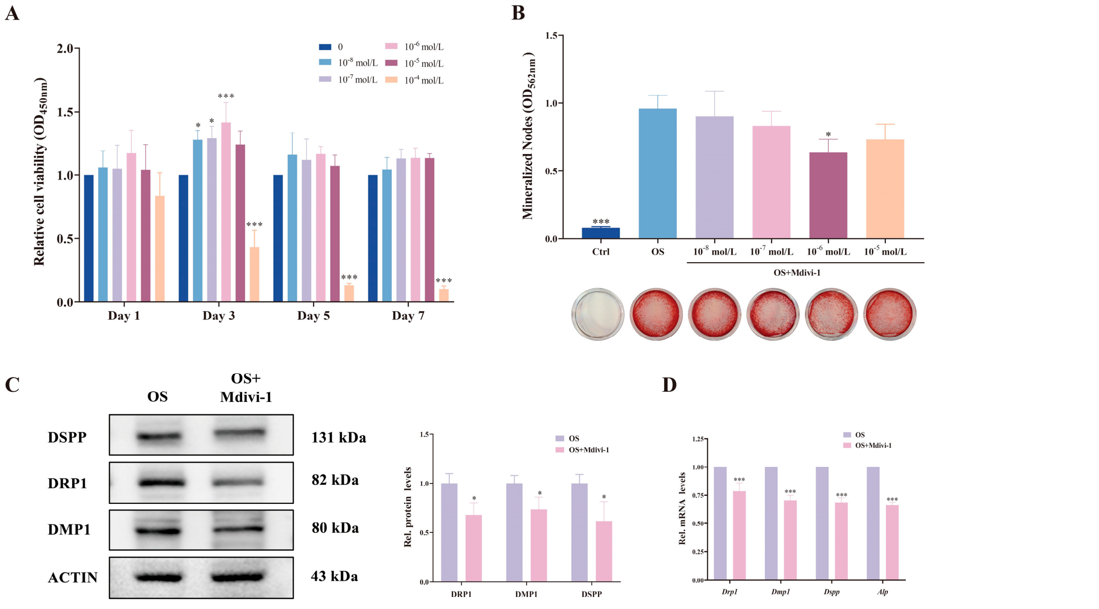

Figure S1. Evaluation of Mdivi-1 effects on DPCs. (**A**) DPCs were cultured with varying concentrations of Mdivi-1 (0, 10^{-4}, 10^{-5}, 10^{-6}, 10^{-7}, 10^{-8} mol/L) for 1, 3, 5, and 7 days. The 10^{-4} mol/L concentration exhibited cytotoxic effects, while 10^{-6}, 10^{-7}, 10^{-8} mol/L concentrations promoted cell proliferation on day 3, with no significant effects observed at other time points compared to the control group (0 mol/L). (**B**) DPCs cultured in osteogenic medium (OS) supplemented with different concentrations (0, 10^{-4}, 10^{-5}, 10^{-6}, 10^{-7}, 10^{-8} mol/L) of Mdivi-1 for 7 days. ARS result showed that treatment with 10^{-6} mol/L Mdivi-1 markedly reduced mineralized nodule formation compared with OS group. Based on its pronounced inhibitory effect on mineralized nodule formation (B) in the absence of cytotoxic effects (A), the concentration of 10^{-6} mol/L Mdivi-1 was selected for further validation. (**C**)Western blot and semi-quantitative analysis of DPCs treated with 10^{-6} mol/L Mdivi-1 during mineralization showed that the protein levels of DSPP, DRP1, and DMP1 were significantly reduced compared with the OS group. (**D**) RT-qPCR analysis of DPCs treated with 10^{-6} mol/L Mdivi-1 during mineralization showed that the relative mRNA levels of *Dspp*, *Drp1*, *Dmp1*, and *Alp* were significantly downregulated. *$p < 0.05$, ***$p < 0.001$.
